# Identification of autoimmune markers in pulmonary tuberculosis

**DOI:** 10.3389/fimmu.2022.1059714

**Published:** 2023-01-25

**Authors:** Anna Starshinova, Anna Malkova, Yulia Zinchenko, Igor Kudryavtsev, Maria Serebriakova, Tatiana Akisheva, Sergey Lapin, Aleksandra Mazing, Dmitry Kudlay, Anzhela Glushkova, Piotr Yablonskiy, Yehuda Shoenfeld

**Affiliations:** ^1^ St. Petersburg State University, St. Petersburg, Russia; ^2^ St. Petersburg Research Institute of Phthisiopulmonology, St. Petersburg, Russia; ^3^ Department of Immunology, Institution of Experimental Medicine, St. Petersburg, Russia; ^4^ St. Petersburg State Medical University, St. Petersburg, Russia; ^5^ First Moscow State Medical University, Moscow, Russia; ^6^ Institute of Immunology, Moscow, Russia; ^7^ Bekhterev Psychoneurological Institute, St. Petersburg, Russia; ^8^ Zabludowicz Center for Autoimmune Diseases, Sheba Medical Center, Tel Hashomer, Israel

**Keywords:** tuberculosis, autoimmunity, trigger-factors, autoantibodies, B-cells, Tfh cells

## Abstract

**Introduction:**

Pathogenesis of many autoimmune diseases is mainly promoted by poorly regulated and/or wrong targeted immune response to pathogens including *M. tuberculosis*. Autoimmunity is one of the processes with are characteristics of tuberculosis (Tbc). The aim was to determine the autoimmune clinical and immunological features in patients with pulmonary Tbc.

**Materials and methods:**

A prospective comparative study was performed in 2017 – 2019 with the inclusion of 46 patients with Tbc. The trigger factors and clinical manifestations, autoantibodies, peripheral blood B cell subsets were stained with fluorochrome-conjugated monoclonal antibodies. 40 healthy volunteers in the control group, were matched for age with no chronic diseases, contacts with TB patients and changes in their laboratory parameters. A statistical analysis was done with GraphPad Prism 6, Statistica 10 (Statsoft) and MedCalc – version 18.2.1 values.

**Results:**

There were no significant ASIA triggers in Tbc patients and control group. 21.1% of Tbc patients had a high level of a rheumatoid factor and in 47.4% complement system factor C3 was high; anti-MCV was detected in 60.7% of Tbc patients. Relative and absolute frequencies of “naïve” Bm1 cells and eBm5 were significantly decreased and activated pre-germinal-center Bm2’ cells were significantly increased in Tbc patients. The CD24++CD38++ B cells were increased in Tbc vs control group (10.25% vs 5.42%), p < 0.001, and 19 cell/1μL (10; 290 vs 11 cell/1μL (6; 20), p = 0.029, respectively). The frequency of CXCR3+CCR4– Tfh1 cells was significantly lower in Tbc vs control one (26.52% vs. 31.00%, p = 0.004), while CXCR3–CCR4+ Tfh2 cells were increased in Tbc (20.31% vs. controls (16.56%, p = 0.030). The absolute numbers of Tfh1 cells were decreased in the Tbc vs. control (24 cell/1μL vs. 37 cell/1μL p = 0.005).

**Conclusion:**

The results of our study showed that the detection of a rheumatoid factor, the components of complement system and anti-MCV in complex with alterations in B cells and follicular Th cell subsets may indicate a presence of autoimmunity in the pathogenesis of tuberculosis, but they are not specific. The indicators of autoimmune-related provide new opportunities in the Tbc treatment.

## Introduction

Currently, tuberculosis (Tbc) remains one of the deadliest infectious diseases worldwide. Approximately, 1.5 mil people died from Tbc in 2020 (1). According to the WHO data, about one-third of the world’s population has a latent Tbc infection, that means people have been infected by *Mycobacterium tuberculosis* (MBT), but they are not with active TB yet. In 5-10% of cases of infected individuals, an active tuberculosis process will develop, while in the other individuals the infection will be latent or asymptomatic (2). The interaction of the MBT with the host organism is poorly studied and may results in the activation or the localization of the infection (3, 4). Despite two decades of an intensified research to understand and cure tuberculosis disease, biological uncertainties remain and hamper the progress. The problem of the spread and treatment of drug-resistant tuberculosis became even more urgent. With the rise of drug resistance, treatment failure rates have increased along with the use of more toxic therapies that are more expencive (5).

According to the WHO, the incidence of multidrug-resistant and extensively drug-resistant tuberculosis increased from 2010 to 2016 (22.0 to 25.8 per 100,000) and tended to decrease only since 2017 (24.7 to 21.4 per 100,000 in 2017 and 2019, respectively) (6, 7). In 2018 484,000 new cases of rifampicin-resistant Tbc were registered worldwide, with 78% of them, being a multiple drug-resistant tuberculosis (MDR-TB). The effectiveness of drug-resistant Tbc treatment does not exceed 56% (7).

Recently, the interest and the research on the autoimmune aspects in tuberculosis have been increasing. It is widely accepted, that the pathogenesis of many autoimmune diseases is mainly promoted by poorly regulated and/or wrong targeted immune response to pathogens, among them MBT (4, 8, 9). Tbc is a multifaceted process having many different outcomes and complications. Autoimmunity is one of the processes characteristics of Tbc. The real meaning of autoantibodies (AAB) in the pathogenesis of Tbc is not quite clear and widely disputed. AAB considered as the result of an imbalanced immune response or as a critical part of the disease pathogenesis. AAB in Tbc might be the markers of comorbid or a provoked autoimmune disease, but there is an alternative point of view regarding them as a protective mechanism helping in the clearance of damaged tissue debris (10, 11).

Previous studies have shown that mycobacteria have antigens similar to human tissues that contribute to the formation of AAB in mycobacterial infection (12). Similar antibodies were found in autoimmune diseases (13). According to many researchers, the development of autoimmune pathology against the MBT infection and the introduction of *M.bovis* with BCG vaccine is associated with a genetic predisposition and features of the immune response (3, 14, 15).

The concept of The autoimmune/inflammatory syndrome induced by adjuvants (ASIA), proposed in 2011 by Shoenfeld Y et al. and the beginning of research on various pathological processes within ASIA, opened new perspectives for understanding of several granulomatosis diseases, for example sarcoidosis, as an autoimmune pathology (16–19).

The autoimmune nature theory of this disease initiated numerous studies (15, 20). In the past few years, vimentinhas gained the attention as a potential autoantigen in some diseases. Vimentin is a cytoskeletal component that presents in the connective tissue and participates in the intercellular interactions and the functioning of the immune system. The occurrence of ААВ to this protein was observed in rheumatoid arthritis, systemic lupus erythematosus and many other connective tissue diseases (20, 21). The presence of autoantibodies in tuberculosis was documented by large amount of studies (22–24).

Some authors suggest that prolonged contact of foreign antigens with toll-like receptors of endotheliocytes, macrophages and dendritic cells lead to activation and epithelioid differentiation of macrophages, which start secreting proinflammatory cytokines in it’s turn (TNF-a, IL-1). Dendritic cells with antigen migrate to the lymph nodes where they present the antigen to T-lymphocytes. After activation, T-lymphocytes differentiate into CD4+, CD8+, Tx17, Treg, proliferate and migrate to the focus of inflammation (23, 24). B cell and humoral immunity play an important part in preventing and rapid effective clearing initial Tbc infection (22, 25).

Most probably, these autoreactive processes may play dual role, both pathogenic and depending on their intensity and specificity. There is no single mechanism of autoimmune inflammation development in Tbc. Several possible mechanisms can be suggested. It may be excessive cell death with impaired clearance of dead cells, impaired autophagy, enhanced activation of macrophages and dendritic cells by MBT as an adjuvant, and also environmental influences, facilitating both Tbc and autoimmune processes in parallels (20, 26). Improved interventions could have a substantial effect on our ability to decrease the morbidity and mortality associated with the disease and limitation further spread, as effective treatment of active Tbc is the major modality for preventing transmission in most of the world.

The identification of the autoimmune characteristics in Tbc patients may be the new step in the understanding of the Tbc inflammation and, in the future, the reasons of the different effectiveness of treatment.

The aim of the study was the determination of the immunological features in patients with pulmonary tuberculosis.

## Materials and methods

### Study population

A prospective comparative study based on the evaluation of clinical and laboratory data with the analysis of sera samples which were collected in 2017 - 2019 at the St. Petersburg Research Institute of Phthisiopulmonology and the City Hospital No. 2.

Exclusion criteria: a period of more than 2 years from the detection of radiographic changes in the lungs, receiving anti-tuberculosis therapy, the presence of HIV infection, syphilis, neoplastic diseases, and decompensated diabetes mellitus.

According to the study design, 46 patients with pulmonary tuberculosis with a bacterial excretion were included (**Table 1**). The control group was comprised of 40 healthy volunteers with no chronic diseases, contacts with tuberculosis and changes in laboratory parameters.

**Table 1:** Demographic, clinical and bacteriological characteristics of Tbc patients.

Characteristics	Tbc patients n (%) (n = 46)
Men	33 (71.7)
Women	13 (28.3)
Age	36.5 (± 10.6) years
Clinical symptoms	41 (89.1)
Fever	25/41 (60.9)
General weakness	31/41 (75.6)
Sweating	21/41 (51.2)
Weight loss up to 5 kg over 5 kg	21 (51.2) 13/21 (61.9) 8/21 (38.1)
Loss of appetite	10/41 (24.4)
Postural tachycardia	2/41 (4.9)
Arthralgia	2/41 (4.9)
Sleep disturbances	2/41 (4.9)
Memory disturbances	2/41 (4.9)
Cognitive impairment	1/41 (2.4)
Respiratory symptoms	
Cough	32/41 (69.6))
Shortness of breath	14/41 (34.1)
Chest pain	9/41 (21.9)
X-Ray symptoms	
Infiltrates in the lungs	15 (32.6)
Focus in the lungs	11 (23.9)
Focal infiltrates and focus in the lungs	20 (25.6)
Pulmonary calcification	7 (15.2)
Fibrotic changes	9 (19.6)
Bacteriologic data	
Sputum positive for MBT	46 (100.0)
Comorbidities	
Endocrine diseases (compensated diabetes mellitus, thyroid pathology)	5 (10.9)
Hepatitis C and B	9 (19.6)
Cardio-vascular pathology	5 (10.9)

According to **Table 1**, clinical manifestations of the disease and comorbidities were detected in 89.1% and in 21.7% of patients respectively.

The study was approved by the Independent Ethical Committee of the St. Petersburg Research Institute of Phthisiopulmonology (protocol No. 34.2 dated 01/19/2017) and the Local Ethical Committee of St. Petersburg State University (protocol No. 01-126 30.06.17). All the participants in the study signed an informed consent.

### Methods

The diagnosis of pulmonary tuberculosis was verified by the *M. tuberculosis* detection in the sputum and/or MBT DNA according to molecular genetics and bacteriological methods, with the presence of typical X-ray changes (27).

### The autoimmune/inflammatory syndrome induced by adjuvants

All study participants were surveyed according to the standardized “ASIA Research Questionnaire”, to assess the impact of ASIA - trigger factors, as well as to determine the compliance of existing clinical manifestations with the ASIA syndrome diagnostic criteria (16). In addition, the history of a professional contact with trigger factors (long-term contact with printer toner, dust, metals, chemicals, cars etc.) before the appearance of clinical and radiological manifestations of the disease were studied (**Table 2**).

**Table 2:** Features evaluated in the “ASIA Research Questionnaire”.

1) Medical history	Autoimmune systemic or organ-specific diseases in the patient and his first-line relatives. Smoking. Allergy to metals, medications, vaccines and others (according to the patient’s history, without special allergy tests). Chronic fatigue syndrome, fibromyalgia, irritable bowel syndrome, history of cancer. Number of pregnancies, duration of breastfeeding. Reception of biological additives, other drugs.
2) Foreign materials	Piercing (including earrings), tattoos, skin fillers (collagen, hyaluronic acid, silicone, etc), silicone implants of any localization, dental amalgam, intrauterine device, contact lenses, heart valves, pacemakers, artificial joints, metal structures, metal implants, dental crowns, veneers - with an assessment of local complications after installation (suppuration, inflammation, necrotic changes, local redness, pruritus), as well as reducing the manifestations of the disease after the removal of foreign materials.
3) Vaccinations received over the past ten years before the onset of the disease	Vaccination (against hepatitis B and A, seasonal influenza, H1N1 influenza, human papillomavirus, DPT vaccine, pneumococcal infection, tetanus vaccine and others); complications from vaccination (within 7 days after vaccination).
4) Clinical manifestations	Fever, general weakness, chronic fatigue, weight loss or weight gain, myalgia, myositis, arthralgia, arthritis, pruritus, chronic rash, peripheral lymphadenopathy, chronic pain, sleep disorders, cognitive impairment, memory disturbances, postural hypotension and tachycardia, recurrent non-infectious cystitis.
5) Biopsy of the involved organ	–
6) Accepted therapy	Analgesics, antihypertensive drugs, sleeping medications, oral contraceptives, aspirin, nonsteroidal anti-inflammatory drugs, hydroxychloroquine, azathioprine, methotrexate, intravenous immunoglobulins, rituximab, tumor necrosis factor inhibitors, corticosteroids and etc.

### Autoantibodies determination

The level of the antibodies to a mutated citrullinated vimentin (anti-MCV) was measured in the sera of 28 patients with Tbc and 40 healthy controls. The patients with positive for anti-MCV antibodies were evaluated for the presence of antibodies to cyclic citrullinated peptide (anti-CCP). Antibodies to anti-MCV were measured using ELISA (ORGENTEC, Germany), anti-CCP with ELISA (Euroimmune, Germany).

Additionally the level of 17 most prevalent autoantibodies were determined. The following antibodies were analyzed: autoantibodies to thyroglobulin (-a-TG), autoantibodies to thyroperoxidase (-a-TPO), IgG antibodies against double-stranded DNA (-dsDNA) were determined using ELISA (Euroimmune, Germany); antinuclear antibodies (-ANA), antibodies to neutrophil cytoplasm (-ANCA), antibodies to smooth muscle (-ASMA), antimitochondrial antibodies (-AMA), antibodies to gastric parietal cells (-APCA) using indirect immunofluorescence (Euroimmune AG, Germany); profile of antinuclear antibodies (SS-a,SS-B,Scl-70, Sm, CENP-B) using immunoblot (Euroimmune, Germany); antibodies to b2-glycoprotein (-b2GP), antibodies to liver and kidney microsomes (-LKM), cardiolipin antibodies IgM, IgG (-ACLA-G, ACLA-M), antibodies to C1q complement factor (-a_C1q) using ELISA (ORGENTEC, Germany).

The manufacturers were used for determination of positive autoantibody concentration.

### Immunophenotyping of B - and follicular Th - cells

**Figure 1:**
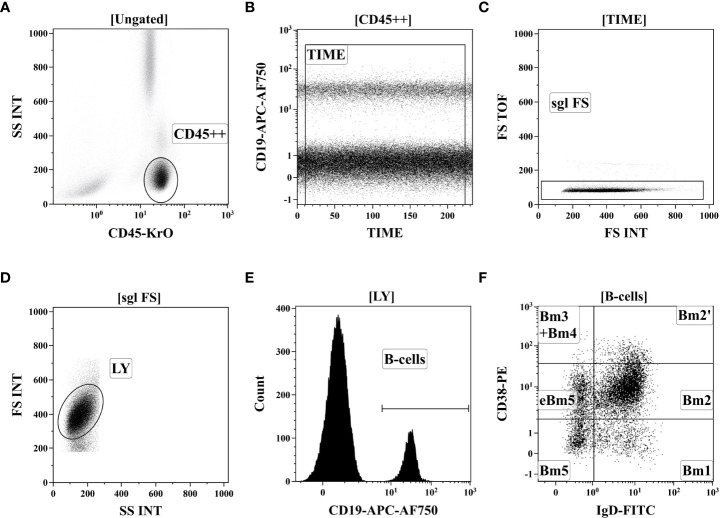
Gating strategy for B-cell immunophenotyping by flow cytometry. **(A)** Total lymphocyte subset identification based on side-scatter and bright CD45 expression. **(B)** Artifact exclusion included by time gating. **(C)** Doublets exclusions from the analysis using FS Int and FS TOF signals ratio. **(D)** Discrimination of lymphocytes and cell debris using FS vs. SS gating. **(E)** B cells were gated as CD19+ lymphocytes. **(F)**. distinct B cell subsets were identified based on of IgD and CD38 co-expression (so-called “Bm1-Bm5” classification): six distinct B-cell subsets were identified, including “virgin naïve” Bm1 cells (IgD+CD38−), “activated naïve” Bm2 cells (IgD+CD38+), pre-germinal-centre Bm2’ cells (IgD+CD38++), common subset, containing centroblasts and centrocytes (marked as “Bm3 + Bm4” cells, IgD–CD38++), and early memory (eBm5) and resting memory cells (Bm5)–IgD–CD38+ and IgD–CD38−, respectively.

200 μL of peripheral blood of 41 Tbc patients and 37 healthy donors was stained for B cell subsets studies with fluorochrome-conjugated monoclonal antibodies: – anti-IgD, anti-CD38, anti-CD183, anti-CD27, anti-CD24, anti-CD19, anti-CD5 and anti-CD45 (25). Red blood cell were lysed, and blood samples were washed by centrifugation and fixed with PBS containing 2% of neutral buffered formalin solution (Sigma-Aldrich),. Sample acquisition was performed using a Navios flow cytometer (Beckman Coulter, Inc.),. At least 5000 CD19+ B cells were analyzed in each sample. Gating strategy was described previously in details (28) and on **Figure 1**.

Flow cytometry data were analyzed by Kaluza software v2.3 (Beckman Coulter, Inc.),. To determine the frequencies of circulating B cell subsets the relative expression of IgD and CD38 (“Bm1-Bm5” classification) was used, which allows to identify IgD+CD38- “naïve” Bm1 cell, IgD+CD38+ “activated naïve” Bm2 cells, IgD+CD38++ pre-germinal-center Bm2’ cells, IgD-CD38++ centroblasts and centrocytes (so-called “Bm3+Bm4” cells), and two subsets of memory B cells – IgD-CD38+ early memory (eBm5) and IgD-CD38- resting memory cells (Bm5) as suggested by Bohnhorst et al. (29).

**Figure 2:**
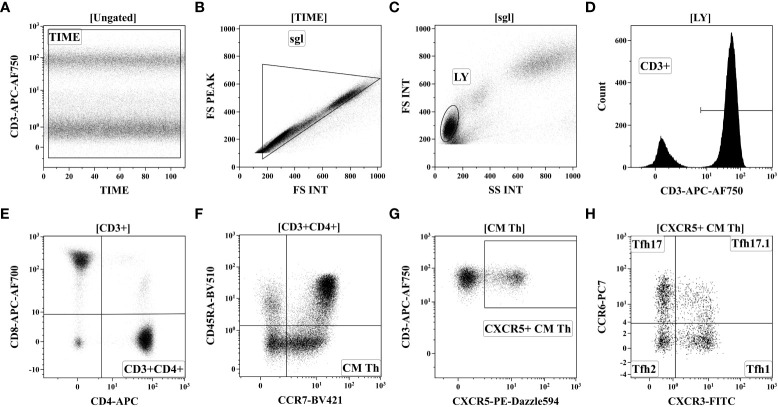
Gating strategy for follicular Th cell subsets immunophenotyping by flow cytometry. **(A)** Artifact exclusion included time gating. **(B)** Doublet exclusions from the analysis using the FS Int and FS Peak signals ratio. **(C)** FS vs. SS gating to discriminate lymphocytes and cell debris. **(D)** Total CD3+ T cell subset identification. **(E)** T-helper (Th) cells gating as CD3+CD4+ lymphocytes. **(F)** Based on CD45RA and CCR7 expression total Th cells were divided into four main subsets, including ‘naïve’ (CCR7+CD45RA+), CM (central memory, CCR7+CD45RA–), EM (effector memory, CCR7-CD45RA−), and TEMRA (T effector memory re-expressing CD45RA, CCR7CD45RA+). **(G)** Within central memory Th cell follicular Th (Tfh) cells were identified based on CXCR5 expression. **(H)** Finally, CXCR5-expressing Tfh cells were classified as Tfh1 (CCR6-CXCR3+), Tfh2 (CCR6-CXCR3−), Tfh17 (CCR6+CXCR3−), and DP Tfh (double-positive Tfh-like, CCR6+CXCR3+).

For follicular Th cell subsets, 200 μL of whole peripheral blood was stained with the following fluorochrome-conjugated monoclonal antibodies cocktail: anti-CD183 (CXCR3), anti-CD25, anti-CD185 (CXCR5), anti-CD194 (CCR4), anti-CD196 (CCR6), anti-CD4, anti-CD8, anti-CD3, anti-CD197 (CCR7) and anti-CD45RA as it was described previously in details (25). The red blood cell were lysed, and blood samples were washed by centrifugation and fixed with PBS containing 2% of neutral buffered formalin solution (Sigma-Aldrich). The sample acquisition was performed using a Navios flow cytometer (Beckman Coulter, Inc., USA). At least 40000 CD3+CD4+ Th cells were analyzed in each sample. Gating strategy was described previously in details (28) and on **Figure 2**.

### Statistical analysis

Statistical analysis was performed using GraphPad Prism 6 (Graph Pad Software), Statistica 10 (Statsoft) and MedCalc – version 18.2.1 (Ostend, Belgium) values. Flow cytometry data were by Kaluza software v2.3 (Beckman Coulter, Inc.),.

The Mann-Whitney-U and Fisher’s exact tests were used for non-parametric data. Quantitative data were presented as m ± sd. The degree of association was calculated using confidence intervals, as well as the χ^2^ test with Yeats correction. To determine the relationship between the values, Spearman correlation analysis was performed. Differences or relationship indicators were considered statistically significant at p-value less than 0.05.

## Results

**Table 3** presents a comparison of the most statistically significant trigger factors in the groups.

**Table 3:** Comparison of the most statistically significant ASIA symptoms and trigger factors in the comparison groups.

ASIA symptoms and trigger factors	Pulmonary tuberculosis n=41	Healthy subjects n=40	χ^2^	P
	n (%)			
ASIA symptoms				
General weakness	31 (75.6)	2 (5.0)	41.8	<0.001
Postural tachycardia	2 (4.9)	0	2.0	p>0.15
Arthralgia	2 (4.9)	0	2.0	p>0.15
Sleep disturbances	2 (4.9)	0	2.0	p>0.15
Memory disturbances	2 (4.9)	0	2.0	p>0.15
Cognitive impairment	1 (2.4)	0	0.9	p>0.3
Postural tachycardia	2 (4.9)	0	2.0	p>0.15
ASIA trigger factors				
Allergy reactions	6 (14.6)	12 (30.0)	0.356	p>0.05
Smoking	12 (29.2)	39 (97.5)	33.81	p<0.001
Piercing (including earrings)	8 (19.5)	4 (10.0)	0.32	p>0.05
Tattoos, Skin fillers (collagen, hyaluronic acid, silicone, etc), Metal structures, Dental amalgam, Metal and silicone implants of any localization	0	0	0	0
Vaccinations received over the past ten years before the onset of the disease	8 (19.5)	12 (30.0)	0.36	p>0.05

The data presented in **Table 3** clearly demonstrate significant differences only of general weakness with significantly higher number of Tbc patients in comparison with healthy subjects (75.6 vs 5.0, χ^2^ = 41.8, p<0.001). Also there were no significant differences in the presence of ASIA triggers in patients with Tbc and in healthy individuals.

B cell maturation was analyzed to assess *M. tuberculosis* infection associated. We compared the frequencies of the main peripheral blood B cell subsets between Tbc patients and the healthy control group. Primarily, we first determined the relative and absolute numbers of peripheral blood CD19+ B cells in the peripheral blood of both groups. No differences in the relative (9.48% (6.35; 15.01) vs 11.09% (9.03; 13.28), p = 0.355) and absolute (179 cell/1μL (122; 321) vs. 228 cell/1μL (161; 287), p = 0.300) were observed between the Tbc patients and the healthy control one.

Next, we determined the frequencies of circulating B cell subsets using the relative expression of IgD and CD38 (“Bm1-Bm5” classification). Using IgD and CD38 staining we were able to identify IgD+CD38- “naïve” Bm1 cell, IgD+CD38+ “activated naïve” Bm2 cells, IgD+CD38++ pre-germinal-center Bm2’ cells, IgD-CD38++ centroblasts and centrocytes (so-called “Bm3+Bm4” cells), and two subsets of memory B cells – IgD-CD38+ early memory (eBm5) and IgD-CD38- resting memory cells (Bm5) as suggested by Bohnhorst et al. (29). The data are summarized in **Table 4**.

**Table 4:** B cell subsets assessed by using “Bm1-Bm5” classification in Tbc patients and healthy subjects.

B cell subset		Tbc patients (n=41) 95%Cl	Healthy subjects (n=37) 95%Cl	Significance
Bm1, IgD+CD38-		9.21 (5.58; 13.99)	14.88 (9.91; 19.13)	p = 0.001
	%(Cl95%)	18.0 (9; 29)	30.0 (18; 50)	p = 0.002
Bm2, IgD+CD38+		59.4 (49,23; 66,83)	53.2 (47.06; 62.6)	p = 0.317
	%(Cl95%)	122 (64; 184)	132 (86; 178)	p = 0.722
Bm2’ IgD+CD38++		11.78 (6.49; 16.21)	4.41 (2.69; 6.45)	p <0.001
	%(Cl95%)	20 (11; 30)	10 (4; 15)	p= 0.001
Bm3+Bm4 IgD-CD38++		0.69 (0.34; 1.16)	0.54 (0.31; 0.94)	p=0.200
	%(Cl95%)	1 (1; 2)	1 (1; 2)	p = 0.768
eBm5 IgD-CD38+		7.31 (5.13; 10.68)	11.56 (8.82; 15.27)	p=0.001
	%(Cl95%)	15 (9; 24)	25 (15; 35)	p=0.004
Bm5 IgD-CD38-		9.42 (4,47; 13,81)	8.62 (6.41;13.74)	p=0.363
	%(Cl95%)	17 (8; 28)	21 (11; 35)	p=0.104

The relative (% within CD19+ cells) and absolute (#, cells/1μL) numbers of B cell subsets assessed by using “Bm1-Bm5” classification in patients with tuberculosis (n = 41) and healthy control (n=37).

We have found that the relative and absolute frequencies of “naïve” Bm1 cells and eBm5 were significantly decreased in patients with tuberculosis compared with the healthy control.

The frequencies of activated pre-germinal-center Bm2’ cells were significantly increased in tuberculosis peripheral blood samples when compared with the healthy control values. Since, we found an increased number of immature transitional Bm2’ cells in patients with tuberculosis, we decided to identify CD24++CD38++ B cells, that expressed immature transitional Bm2’ cells, but they were able to produce IL-10 (30).

We have proven that in Tbc patients increased relatively and absolute numbers of CD24++CD38++ B cell in their peripheral blood vs. the health control group(10.25% (5.64; 15.47) vs. 5.42% (3.38; 7.42), p < 0.001, and 19 cell/1μL (10; 290 vs. 11 cell/1μL (6; 20), p = 0.029, respectively).

Firstly, we addressed the analysis of follicular T-helper cells (Tfh) that control all stages of B cell maturation and facilitate antibody responses to viral, bacterial, parasite, and fungal infections (31). It is known that peripheral blood circulating Tfh cells show a CD45RA–CCR7+ “central memory phenotype” and express chemokine receptor CXCR5, allowing Tfh cells for migration to the T-B border in secondary lymphoid organs of different location. Finally, the differences in the relative (35.28% (28.19; 40.86) vs. 32.33% (28.28; 36.61), p = 0.270) and absolute (95 cell/1μL (53; 130) vs. 120 cell/1μL (82; 159), p = 0.062) numbers were observed between the patients with pulmonary tuberculosis and healthy controls.

For identifying the distinct Tfh cell subsets we analyzed the chemokine receptors CXCR3 and CCR6 co-expression on central memory Tfh cells

Previously, Morita et al. shown that the total circulating Tfh cell compartment could be divided into CXCR3+CCR6− Tfh1-like, CXCR3−CCR6− Tfh2-like, CXCR3−CCR6+ Tfh17-like, and ‘double-positive’ CXCR3+CCR6+ Tfh cells (32).

**Figure 3:**
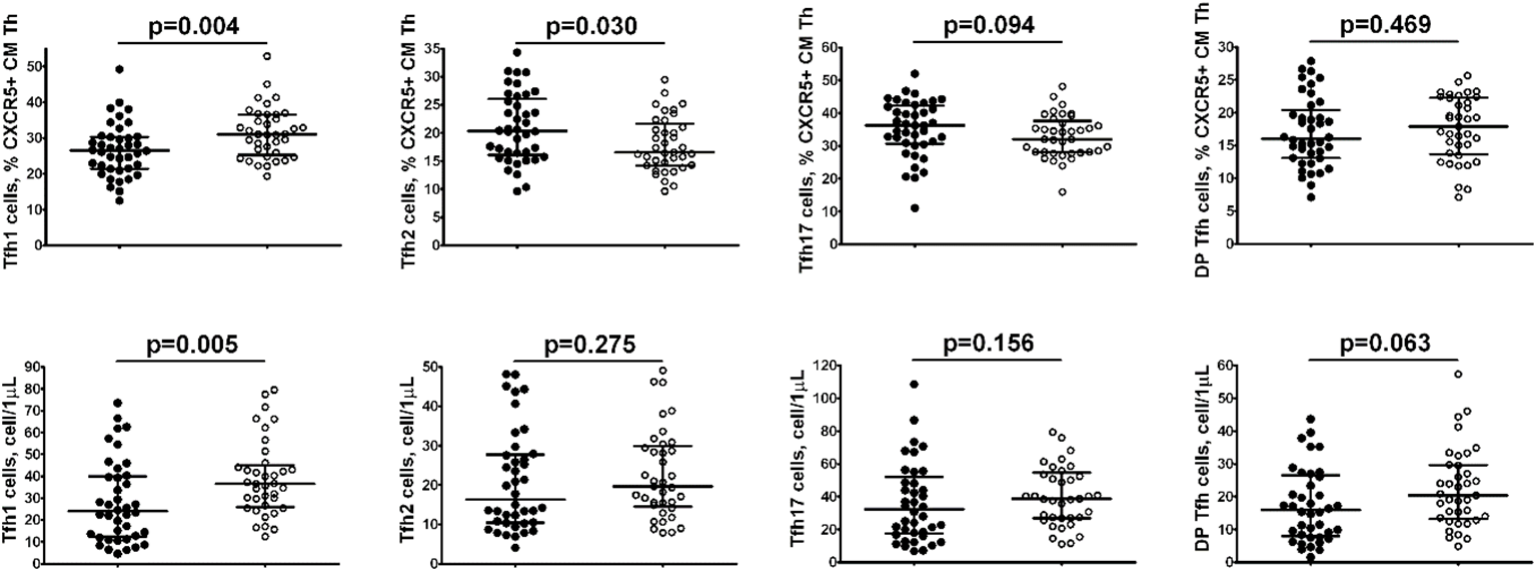
Comparison of relative numbers of main central memory Tfh cell subsets between Tbc patients.

Comparison of relative numbers of main central memory Tfh cell subsets between Tbc patients and the healthy control group (**Figure 3**), indicated a significantly lower frequency of CXCR3+CCR4– Tfh1 cells in *M. tuberculosis* infection group (26.52% (21.43; 30.32) vs. 31.00% (25.85; 36.53), p = 0.004), while CXCR3–CCR4+ Tfh2 cells were increased (20.31% (16.06; 26.07) vs. 16.56% (14.27; 21.00), p = 0.030).

Furthermore, the absolute numbers of Tfh1 cells also decreased in patients with tuberculosis vs the control group (24 cell/1μL (12; 40) vs. 37 cell/1μL (27; 44), p = 0.005).

The level of autoantibodies to a modified citrullinated vimentin (anti-MCV) and to cyclic citrullinated peptide (anti-CCP) were additionally determined in Tbc patients (**Table 5**). According to the data presented in **Table 5**, anti-MCV was detected in 60.7% (17/28) of patients with tuberculosis, which was significantly more often than in the control group (25.0%). The level of anti-CCP was low.

**Table 5:** The levels of anti-MCV and anti-CCP autoantibodies in the sera of the participants.

Study Groups	Results of anti-MCV testing		CI 95%	Results of anti -CCP		CI 95%
	An increased level n/%	Absolute value Med		An increased level n/%	Absolute value Med (Q25; Q75)	
Pulmonary tuberculosis n=28	60.7* (17/28)	23.39	19.17-27.61	0 0/17	2.26	0.96-4.55
Healthy subjects n=40	25.0 (10/40)	14.75	11.52-17.99	0 (0/10)	0.55	0.87-2.10

*р<0.01 – a significant difference between the values in tuberculosis and in the control group.

The spectrum of most prevalent antibodies is presented in **Table 6**.

**Table 6:** Titer of antibodies in Tbc patients.

Antibodies	Reference values	Tbc patients n=19		Healthy subjects n=25	
		abs. (M; 95%Cl)	High level of autoantibodies (n/%)	abs. (M; 95%Cl)	High level of autoantibodies (n/%)
Anti-TPO, IU/ml	N<50	47.5; 4.92 – 364.42	2 (10.5)	35.5; 2.82 – 264.48	1 (4.0)
Anti-Cardiolipin CL G, U/ml	N<10	1.68; 0.92-3.04	0	0.58; 0.32-2.04	0
Anti-Cardiolipin CL M, U/ml	N<10	2.26; 0.95 – 11.71	1 (5.2)	1.16; 0.55 – 10.51	1 (4.0)
Anti-Нер-2 cellular antigens	1:1280-40000 - high	168.8; 160-320	1 (5.4)	188.9; 172-470	0
Anti-neutrophil cytoplasmic (ANCA)	>1:40 - positive	40.0	0	30.0	0
Anti-beta2-glycoprotein, B2GP Total, RU/ml,	N<20	20.34; 0.2-156.81	3 (15.7)	15.64; 0.12-186.0	2 (8.0)
Anti-thyroglobulin TG, IU/ml,	N<100	161.25; 2.15-2.423	2 (10.5)	92.34; 1.14-1.643	0
Anti-DNA, IU/ml,	N<25	3.13; 0.1-56.12	1 (5.2)	6.18 0.2-26.14	0
Anti-Saccharomyces cerevisiae Ig, GASCA G, RU/ml,	N<20	13.51; 0.64-86.29	3 (15.8)	8.53; 0.54-43.22	0
Anti-Saccharomyces cerevisiae IgА, ASCA A, RU/ml,	N<20	2.26; 0.95-11.71	0	12.36; 1.97-13.75	0
Rheumatoid factor, RF, IU/ml,	N<20	27.42; 20-144	4 * (21.1)	15.34; 15-122	0
Complement factor level C3, C3, g/l,	N 0,75-1,65	1.16; 0.98-2.81	9* (47.4)	0.34 0.45-1.51	2 (8.0)
Complement factor level C4, C4, g/l,	N 0,13-0,54	0.40; 0.17-0.6	3 (15.8)	0.32; 0.15-0.45	1 (4.0)
Anti- mitochondrial (АМА)	<1:40	40	0	40	0
Liver-kidney microsomes (LKM) antigens	<1:40	40	0	40	0
Smooth muscle antigens (SMA)	<1:40	40	2 (3.6)	40	0
Gastric Parietal Cell (GPC) antigens	<1:40	40	0	40	0

*р<0.05 – a significant difference between the values in tuberculosis and in the control group.

No significant differences in the detection of the antibodies in the comparison groups were observed. However, 21.1% of tuberculosis patients had a high level of a rheumatoid factor and in 47.4% the complement system factor C3 was high in Tbc patients (**Table 4**).

## Discussion

To date, the diagnosis and treatment of tuberculosis infection remains a problem for the world community. Vaccination with the use of BCG, the use of new drugs did not allow coping with the annual spread of infection and the formation of drug-resistant forms of tuberculosis.

Studies of the autoimmune response in Tbc have been conducted since the middle of the XIX century. Many scientists note the presence of clinical symptoms of autoimmune diseases in tuberculosis patients, the appearance of autoantibodies, the presence of a genetic predisposition (4, 33–35). The existing assumptions have not yet found unambiguous evidence of the autoimmune inflammation in tuberculosis and its effect on the course of the disease, but research in this direction continues.

Our study is an attempt of searching for autoimmune characteristic in patients with pulmonary tuberculosis by methods currently used in diagnostic of autoimmune diseases.

### Tuberculosis and autoimmunity

Previously, the relationship between the development of autoimmune pathology after the introduction of an attenuated strain of *M. bovis* was shown. *M. bovis* is the main causative agent of tuberculosis in cattle and it is used for immunization in humans to date, both for the prevention of Tbc and for the treatment of oncological pathology and even severe COVID-19 (8, 26, 35, 36).

In the experiment, Mtb is quite often used as an adjuvant, for example, in a complete Freund adjuvant in animal models of autoimmune diseases (37), which is presumably related to the fact that these antigens overcome tolerance to host antigens when co-administered. In experimental models, Mtb immunization can cause autoimmune joint lesions by the cross-reactivity with proteoglycan in cartilage (35).

Currently, there is an evidence of the trigger role of *M. tuberculosis* in the development of diseases such as sarcoidosis, systemic lupus erythematosus, rheumatoid arthritis, primary biliary cirrhosis and many others (11, 19, 38–40).

### Autoantibodies in tuberculosis

No reliably known mechanism which are responsible for the formation of antibodies in tuberculosis. This antibodies have been identified to date. There are assumptions about possible mimicry between the antigenic structure of mycobacteria and the host tissue’s own antigens (12, 21, 41).

At the same time, a number of studies have shown that in 40% of cases in tuberculosis patients, antibodies typical for granulomatosis with polyangiitis, systemic lupus erythematosus and other autoimmune diseases were detected (4, 35). Statistically significant increase in plasma concentrations of antibodies in tuberculosis patients was diagnosed to ribonucleoproteins (15%), anti-SSA (64%) and anti-ACA-IgM antibodies (59%) (42–44). In rare cases, antibodies to neutrophil cytoplasm, antibodies to beta-2-glycoprotein (anti-b2GPI), antibodies to cyclic citrullinated peptide, anticardiolipin antibodies are registered. At the same time, the frequency of detection of these autoantibodies was in some cases similar to that in patients with autoimmune pathology, as well as anti-tuberculosis treatment can lead to normalization of some AAB (35, 45, 46).

### T and B-cells lymphocytes

Previously, Elkholy et al. reported that the frequency of CD3-CD19+ B cell in peripheral blood from patients with active pulmonary tuberculosis was significantly lower than in a control group (47). In contrast, Wu et al. found that CD19+ B cells were increased in Tbc patients vs a control group (48). We noticed no differences in relative and absolute numbers on total CD19+ B cells subset between *M. tuberculosis* infected patients and a healthy control, but we found dramatic alterations in B cell subsets composition.

Our data indicated that peripheral blood B cells from Tcb patients showed the decreased levels of “naïve’ B cell that expressed unique B cell receptors and were able to recognize and to initiate immune response to new specific antigens in secondary lymphoid organs. Furthermore, *M. tuberculosis* infected patients had low levels of memory eB5 cells, that was essential for long-term protection against reinfection. Thus, the recognition of new antigens and effective activation of immunological memory which have been encountered previously and they could functionally impaired during *M. tuberculosis* infection. Similarly, Joosten et al. reported that in Tbc patients the level of atypical circulating subsets (CD21−CD27− or IgD−CD27−) and activated (IgD−CD27+) B-cellsreduced numbers of IgD+CD27− naïve B-cells if compared to control (49). Furthermore, we found in Tbc patients IgD+CD38++ pre-germinal-center Bm2’ cells were activated, that could leave the blood stream, enter secondary lymphoid organs and initiate humoral immunity in response to antigen recognition. We should mention that Bm2’ cells are highly heterogeneous, and may contain regulatory B cell subset with CD24++CD38++ phenotype, as it was shown previously by Blair et al. (50). Similarly, several paper also reported that peripheral blood circulating Bregs with CD19+CD1d+CD5+ phenotype were increased in patients with tuberculosis (51, 52). Interestingly, Breg subsets were found and increased in patients with various autoimmune diseases, including ankylosing spondylitis (53), primary Sjögren’s syndrome (54), diabetes mellitus (55), multiple sclerosis and neuromyelitis optica spectrum disorders (56), etc., suggesting a close involvement of CD24++CD38++ B cells in the autoimmune pathogenesis.

Tfh cells have been shown to mediate protective immunity against tuberculosis *via* accumulation in infected lungs and production of proinflammatory cytokines, while mice deficient in Cxcr5 had increased susceptibility to Tbc due to defective T cell localization within the lung parenchyma (57). Recently, Sun et al. noted that the frequencies of Tfh cells with the following phenotypes CD4+CXCR5+, CD4+CXCR5+ICOS+ and CD4+CXCR5+PD-1+Tfh as well as peripheral blood IL-21 levels were increased in Tbc patients if compared to healthy controls (58). Controversially, Kumar et al. observed that active pulmonary tuberculosis was characterized by diminished frequencies of Tfh cells ex vivo and in response to Tbc antigens and by diminished frequencies Tfh cells producing IL-21 (23). Though, we found no differences in circulating memory Tfh cells between *M. tuberculosis* infected patients and healthy controls, however, we noticed alterations in Tfh cell subsets. However, the absolute number and the frequency of Th1 cells within the CD45RA–CCR7+CXCR5+ CD4+ T cells compartment were significantly lower in patients with tuberculosis when compared to a healthy control, while the relative number of Tfh2 cell was increased. Indeed, imbalance of cytokines that were realized in the site of *M. tuberculosis* infection and were produced by activated dendritic cells in secondary lymphoid tissue could dramatically influence Th cell polarization and affect all types of Th cell subsets, including different subsets of Tfh cells (59).

CXCR3+ Tfh1 diminished the functions to provide help to B cells in a mouse malarial model (60) and human *in vitro* experiments, when they were shown to induce apoptosis in activated naïve B cells (61), while Tfh2 and Tfh17 induced differentiation of naive B cells to plasma cells, activated class-switching and stimulated antibodies production (32). Thus, the ratio of Tfh2 and Tfh17 that provided help to B cells over Th1 (able to block B cell activation) was considered to be important for effective humoral immune responses. Furthermore, numerous studies also suggested that an altered balance of blood CXCR5+ CD4+ Tfh cell subsets was closely linked with autoimmunity. For instance, in SLE patients with an active Tbc (SLEDAI score>8) the frequency of Tfh2 was significantly increased that was accompanied with Tfh1 cell subset percentage decrement, and alterations in relative numbers of Tfh2 and Tfh1 cells were associated with the presence of high Ig levels and autoantibodies in patient’s sera (62). Furthermore, patients with relapsing-remitting and secondary progressive multiply sclerosis decreased frequencies of Th1-like Tfh cells (63). Next, patients with IgG4-related disease exhibited significantly the increased level of Tfh2 cells and significantly the decreased level of Tfh1 cells compared to a healthy control group (64). Thereby, altered Tfh subset balance was associated with a wide range of autoimmune diseases in humans, but still it is poorly study.

The anti-MCV antibodies were defined in Tcb patients and not in a control group, that could imply an autoimmune response in pathogenesis of the disease (65–68).

The presence of an autoimmune component associated with an increase in the level of autoantibodies may be significant for the correction of therapy and serves as a criterion for considering the appointment of immunosuppressive therapy in the future.

Probably, the reason for the elevation of autoantibodies may be molecular mimicry, which is described in chronic infections with a hyperactive immune response. Those findings are coherent with our data. The heat shock proteins Mtb-HsP60, Mtb-HsP65, catalase (mKatG) can be considered as the candidate mycobacterial antigens, possibly involved in the cross-reaction (69–71).

The presence of antibodies in patients with tuberculosis may reflect the relationship between the pathogenesis of those diseases with the possibility of cross-reactivity between vimentin and *M. tuberculosis* peptides (71–73).

## Conclusion

According to the presented data, some immunological parameters could be an identification of an autoimmune component in the pathogenesis of tuberculosis. Alterations in B cells and follicular Th cell subsets indicate autoimmune-related pathways in pathogenesis of *M. tuberculosis* infection and provide opportunities in pulmonary tuberculosis treatment. The category of patients with the alteration of the balance of T and B lymphocytes requires further examination of the level of antibodies. We have shown that among the large spectrum of antibodies only some of them may require attention in Tbc (RF, components of the complement system, anti-MCV).

When determining the symptoms characteristic of autoimmune diseases, only chronic fatigue was significantly determined in Tbc patients. The analysis of ASIA triggers also did not show significant differences in Tcb patients.However, it should be remembered that *M. tuberculosis* itself is a trigger that provokes the development of an autoimmune reaction with a specific orientation of the cellular immune response, but only in 50% of cases.

The obtained results require further study, especially the determination of the level of autoantibodies after the anti-tuberculosis therapy in patients with a different spectrum of sensitivity of mycobacteria. In the future, determining the parameters of autoimmune inflammation and its correction at the stage of prescribing therapy in Tbc may be a key point in improving the efficacy of treatment, the absence of progression of the Tbc process and preventing the formation of drug resistance.

## Data availability statement

The original contributions presented in the study are included in the article/Supplementary Material. Further inquiries can be directed to the corresponding author.

## Author contributions

Formal analysis: YZ. Investigation: DK. Methodology: AnM, YZ, SL and AlM. Project administration: AS, DK, PY and YS. Validation: AnM. Writing—original draft: AS, YZ and AG. Writing—review and editing: YS. All authors have read and agreed to the published version of the manuscript. All authors contributed to the article and approved the submitted version.
